# Fast Polymeric Functionalization Approach for the Covalent Coating of MoS_2_ Layers

**DOI:** 10.1021/acsami.1c08294

**Published:** 2021-07-23

**Authors:** Iván Gómez-Muñoz, Sofiane Laghouati, Ramón Torres-Cavanillas, Marc Morant-Giner, Natalia V. Vassilyeva, Alicia Forment-Aliaga, Mónica Giménez-Marqués

**Affiliations:** Instituto de Ciencia Molecular (ICMol), Universidad de Valencia, c/Catedrático José Beltrán 2, Paterna 46980, Spain

**Keywords:** 2D materials, transition metal dichalcogenides, covalent functionalization, diazonium chemistry, surface polymerization

## Abstract

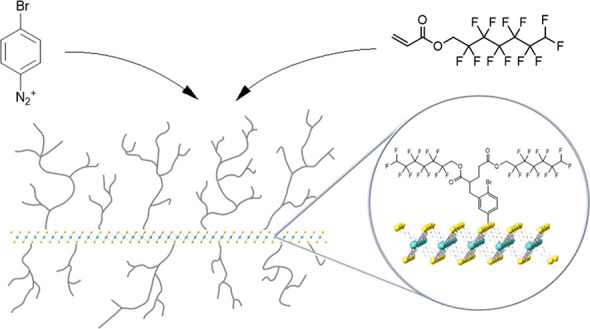

We present the covalent coating of chemically exfoliated molybdenum disulfide (MoS_2_) based on the polymerization of functional acryl molecules. The method relies on the efficient diazonium anchoring reaction to provoke the in situ radical polymerization and covalent adhesion of functional coatings. In particular, we successfully implement hydrophobicity on the exfoliated MoS_2_ in a direct, fast, and quantitative synthetic approach. The covalent functionalization is proved by multiple techniques including X-ray photoelectron spectroscopy and TGA-MS. This approach represents a simple and general protocol to reach dense and homogeneous functional coatings on 2D materials.

## Introduction

Transition metal dichalcogenides (TMDCs) represent one of the most studied families of lamellar compounds that can be easily exfoliated into two-dimensional (2D) layers, exhibiting a plethora of unique physical and chemical properties.^1^ These 2D materials display an MX_2_ stoichiometry (where M is a transition metal and X is sulfur, selenium, or tellurium) and, depending on their structural arrangement, distinct polytypes with completely different catalytic, magnetic or electronic properties can be obtained.^2−6^ These physical features make TMDCs very attractive for their integration in 2D-based nanotechnologies such as optoelectronics or sensing.^7,8^

MoS_2_ is undoubtedly the flagship of the TMDC family because of its scalable preparation through simple exfoliation methods and amenable functionalization through chemical design.^9,10^ The molecular functionalization of MoS_2_ has been extensively explored to induce changes in its physical and mechanical properties,^11,12^ modify its processability,^13^ or even add new functionalities.^14−16^ Different methods mainly involving electrostatic^17^ and/or covalent functionalization have been used to randomly distribute molecules on the surface,^14,18−22^ the latter being generally preferred to ensure the chemical robustness of the MoS_2_ functionalized system.

Among surface covalent strategies, the chemistry of aryl radicals is particularly interesting, as it occurs even in the absence of edge sites or defects.^14^ However, in the particular case of MoS_2_ covalent functionalization based on diazonium, a limited density of attached molecules is often reached (∼11% coverage), which is marked by the number of molecules activated by CE-MoS2 as reducing agent^19^. This surface density has been recently improved by the addition of supplementary reducing agents such as KI, which promotes the massive reduction of aryl diazonium salts in solution, leading to practically overall surface coverage.^23^ A suitable alternative to promote considerably MoS_2_ functionalization is the use of reactive molecules capable of fostering in situ polymerization. This polymeric approach leads to the formation of a dense coating in the final material, which results ideally in effectively implementing a desired functionality by selecting the appropriate functional monomer. Several examples have been reported, including polymerization on graphene through imine-based chemistry^24^ and more recently in MoS_2_ using maleimides^18^ and poly(*N*-vinylcarbazole).^25^ However, apart from these examples, methods to develop polymeric functional coatings on MoS_2_ remain scarce.

**Figure 1:**
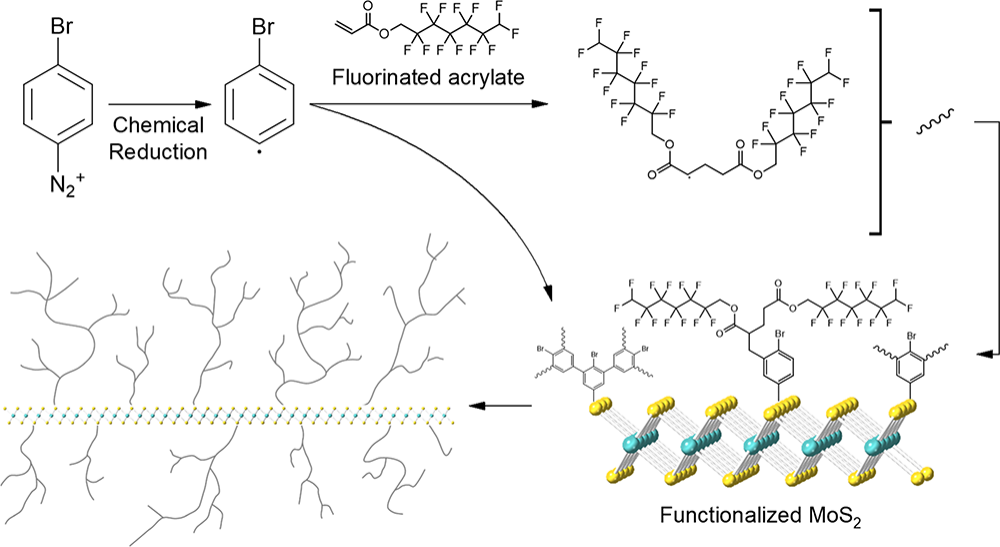
Schematic representation of the proposed polymeric reaction on MoS_2_.

Herein, we present a versatile polymeric reaction that provides CE-MoS_2_ with a desired functional shell. We take advantage of the robust covalent archetypal diazonium grafting occurring via aryl radicals to promote the polymerization of functional vinyl monomers. As a result, CE-MoS_2_ is coated with a large number of functional moieties, regardless of the extent of molecules directly anchored to the surface (Figure 1). The synthesis is adapted from a grafting method^26^ that has been applied to a large number of materials with distinct nature such as carbon nanotubes^27^ and fibers,^28^ metal surfaces or metal oxide nanoparticles,^29^ and recently to porous metal–organic frameworks,^30,31^ but to the best of our knowledge has not been used in TMDCs. This covalent surface reaction is particularly convenient since it occurs under fast mild conditions in a straightforward manner, leading to the anchoring of large amounts of molecules with the desired functionality.

In this work, we selected the in situ formation of hydrophobic coatings, which serve as the basis to develop air-stable and more processable functional polymer-coated 2D materials.

## Results and Discussion

The quantitative grafting of functional polymers using a diazonium anchoring process was first adapted to functionalize MoS_2_ flakes (see Figure 1). The grafting process consisted of directly mixing a suspension of MoS_2_ flakes with an aryl diazonium salt in the presence of acrylate monomers. Two fluorinated acrylate monomers of different chain lengths were explored in parallel reactions, namely, 1,1,1,3,3,3-hexafluoroisopropyl acrylate (acryl-C_3_F_6_) and 2,2,3,3,4,4,5,5,6,6,7,7-dodecafluoroheptyl acrylate (acryl-C_7_F_12_). An aqueous colloid of CE-MoS_2_ was first obtained following a previously reported method based on the intercalation of *n*-BuLi^12^ (see Experimental Section for further details and Figures S1–S3). The resulting flakes exhibit a negative surface charge, as measured by the ζ-potential of aqueous suspensions. This chemical characteristic is expected to increase the reactivity of their basal plane. Two exfoliated suspensions were mixed in parallel reactions with an acetonitrile solution containing the aryl diazonium salt in a 1:3 molar ratio (MoS_2_:bromobenzene diazonium). Then, an excess of the corresponding fluorinated acrylate molecules (10 equiv. of MoS_2_-based) were immediately added to the mixtures under vigorous stirring in air. The reactions were instantaneously initiated upon the addition of reagents and completed within a few seconds, as deduced by the fast formation of a black flocculate (see Figure S4). Essentially, the reaction is initiated by the well-known formation of aryl radicals that directly graft to the MoS_2_ surface forming a phenylene layer, as described previously.^32^ Simultaneously, the radical polymerization of the different fluorinated acryl-C_3_F_6_ and acryl-C_7_F_12_ monomers occurs in situ to form polymeric structures that covalently attach to the phenylene layer. As a result, the quantitative grafting of fluorinated molecules in the form of a polymer film is covalently anchored to the MoS_2_ surface using a mild radical-based reaction. As a control reaction, a mixture of MoS_2_ flakes and vinyl monomers was prepared in the absence of the diazonium molecules, leading to the uncoated material. This control experiment confirms the dual role of the diazonium salt that, in the presence of CE-MoS_2_ as reducing agent takes up an electron leading to aryl radicals^33^ that (i) link to the MoS_2_ basal-plane to form a phenylene layer and (ii) initiate the radical polymerization of the vinylic monomers in solution. After centrifuging and thoroughly washing off the black solids obtained, the functionalized MoS_2_ compounds, hereafter MoS_2_@C_3_F_6_ and MoS_2_@C_7_F_12_, were isolated and characterized.

**Figure 2:**
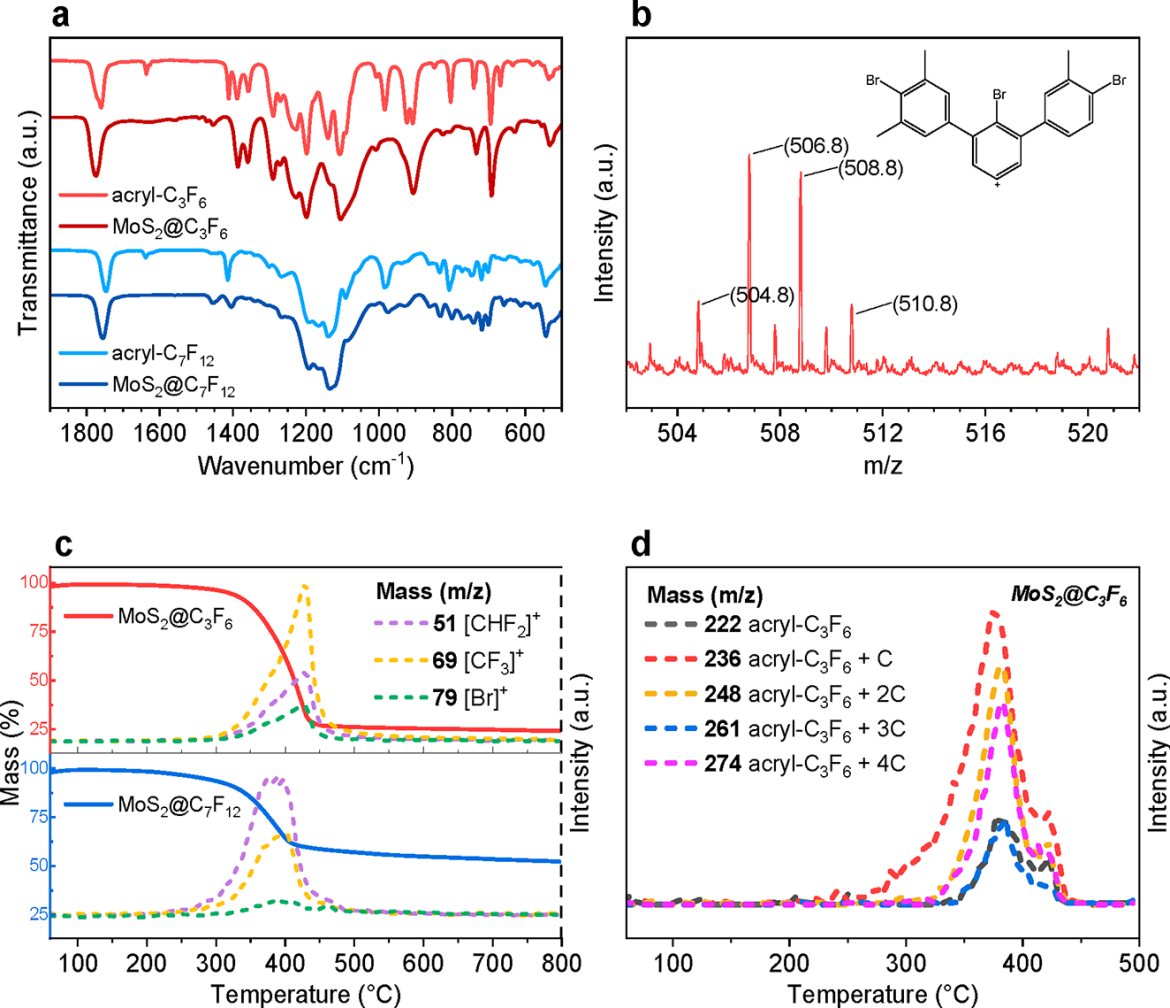
(a) Infrared spectra of functionalized MoS_2_@C_3_F_6_ (dark red) and MoS_2_@C_7_F_12_ (dark blue) materials as compared to corresponding commercial fluorinated acryl monomers (light colors). (b) Isotopic distribution of the base peak of MALDI-TOF measurements attributed to three covalently bonded bromoaryl molecules. (c) Thermal profiles of functionalized MoS_2_@C_3_F_6_ (red) and MoS_2_@C_7_F_12_ (blue) materials with the corresponding coupled mass selected peaks detected upon thermal treatment as deduced from TGA-MS spectrometry. Molecular moieties detached correspond to CHF_2_ (purple), CF_3_ (yellow), and Br (green). (d) In-depth analysis of the mass fragments detached upon thermal treatment in the MoS_2_@C_3_F_6_-coated material. The selected molecular fragments correspond to successive carbon additions and match with a vinylic polymer formation.

MoS_2_ polymer functionalization was evidenced by the feasible dispersibility and colloidal stability of MoS_2_@C_3_F_6_ and MoS_2_@C_7_F_12_ in acetonitrile and dichloromethane, which contrasts with the colloidal instability of MoS_2_ under similar conditions. We first analyzed the functionalization by infrared spectroscopy. Figure 2a depicts the ATR-FTIR spectra of the corresponding functionalized MoS_2_@C_3_F_6_ and MoS_2_@C_7_F_12_ materials compared to the commercial fluorinated molecules. The two pairs of spectra present similar bands arising from the fluorinated molecules, with particularly relevant differences. In the functionalized MoS_2_, the band at 1635 cm^–1^ attributed to the acryl vibration (ν C=C) disappears,^34^ in agreement with a grafting reaction. This loss of the C=C functionality is also confirmed by a shifting of the intense band at ∼1740 cm^–1^ attributed to the C=O vibration toward higher energies, consistent with the transformation of an α,β-unsaturated ketone to a saturated one (Figure S5), as well as the loss of the band at ∼1410 cm^–1^, assigned to the C–H vibration in C=C.^35^ In addition, the absence of the band at 2286 cm^–1^ attributed to the N–N vibration of the N_2_^+^ group in the diazonium salt^36−38^ confirms its elimination (Figure S6). The effect of the polymer grafting on CE-MoS_2_ was then studied by Raman spectroscopy, where the characteristic J peaks (154, 226, and 330 cm^–1^) of CE-MoS_2_ are not detectable upon functionalization, likely due to the presence of a thick polymeric layer that hinders their characterization (Figure S7).

Thermogravimetric analyses of the functionalized MoS_2_@C_3_F_6_ and MoS_2_@C_7_F_12_ as compared to the CE-MoS_2_ were analyzed to estimate the degree of coverage. Profiles depicted in Figure 2c for the functionalized MoS_2_@C_3_F_6_ and MoS_2_@C_7_F_12_ show no significant mass loss below 300 °C, which is followed by a weight loss in the temperature range 300–500 °C equivalent to 76 and 48%, respectively, for MoS_2_@C_3_F_6_ and MoS_2_@C_7_F_12_. These profiles contrast with the typically large thermal stability associated with CE-MoS_2_ (see Figure S3) and should be attributed to the thermal decomposition of the grafted organic coating (Figure S8). Remarkably, the obtained large mass loss corresponding to a significant presence of organic coating contrasts with the typically moderate organic content obtained in truly molecular surface functionalization previously reported.^39,40^ This evidence supports the formation of a branched-like polymer film in MoS_2_@C_3_F_6_ and MoS_2_@C_7_F_12_ materials as illustrated in Figure 1. In an attempt to elucidate the moieties thermally detached from the functional hybrid coated material, we coupled thermogravimetric analysis to mass spectrometry (TGA-MS) (Figure 2c, d). Three main mass peaks with *m*/*z* = 51, 69, and 79 respectively attributed to CF_2_ and CF_3_ groups and a Br moiety were detected at 430 and 400 °C, respectively, for MoS_2_@C_3_F_6_ and MoS_2_@C_7_F_12_ materials (Figure 2c), which confirms the presence of fluorinated monomers and bromoaryl molecules in the coating shell. A more detailed analysis in the MoS_2_@C_3_F_6_ material evidence mass losses peaks with larger *m*/*z* values, mainly *m*/*z* = 222, 236, 248, 261, and 274 (Figure 2d), which are attributed to successive additions of one carbon-chain fragment to a vinyl monomer (see Figure S9 for further details). These detected molecular fragments support the formation of the covalent coating via an induced radical polymerization of the acrylic monomers to form a fluorinated polymeric shell.

MALDI-TOF spectrometry was then used to gain insight into the surface functionalization mechanism (Figure 2b and Figure S10). It was found that the base peak of the spectrum appears at *m*/*z* = (505, 507, 509, 511), which is assigned to a molecular fragment comprising three covalently bonded bromoaryl molecules, as deduced from isotopic distribution. The detection of this large molecular fragment is in agreement with the further aryl radical attack to pregrafted aryl species, supporting the idea that the diazonium reaction proceeds by a radical mechanism. As a result, a polyphenylene sublayer is directly anchored onto the MoS_2_ basal plane, acting as a covalent bridge between the MoS_2_ and the polymeric fluorinated coating, as previously reported.^27,29−31^

**Figure 3:**
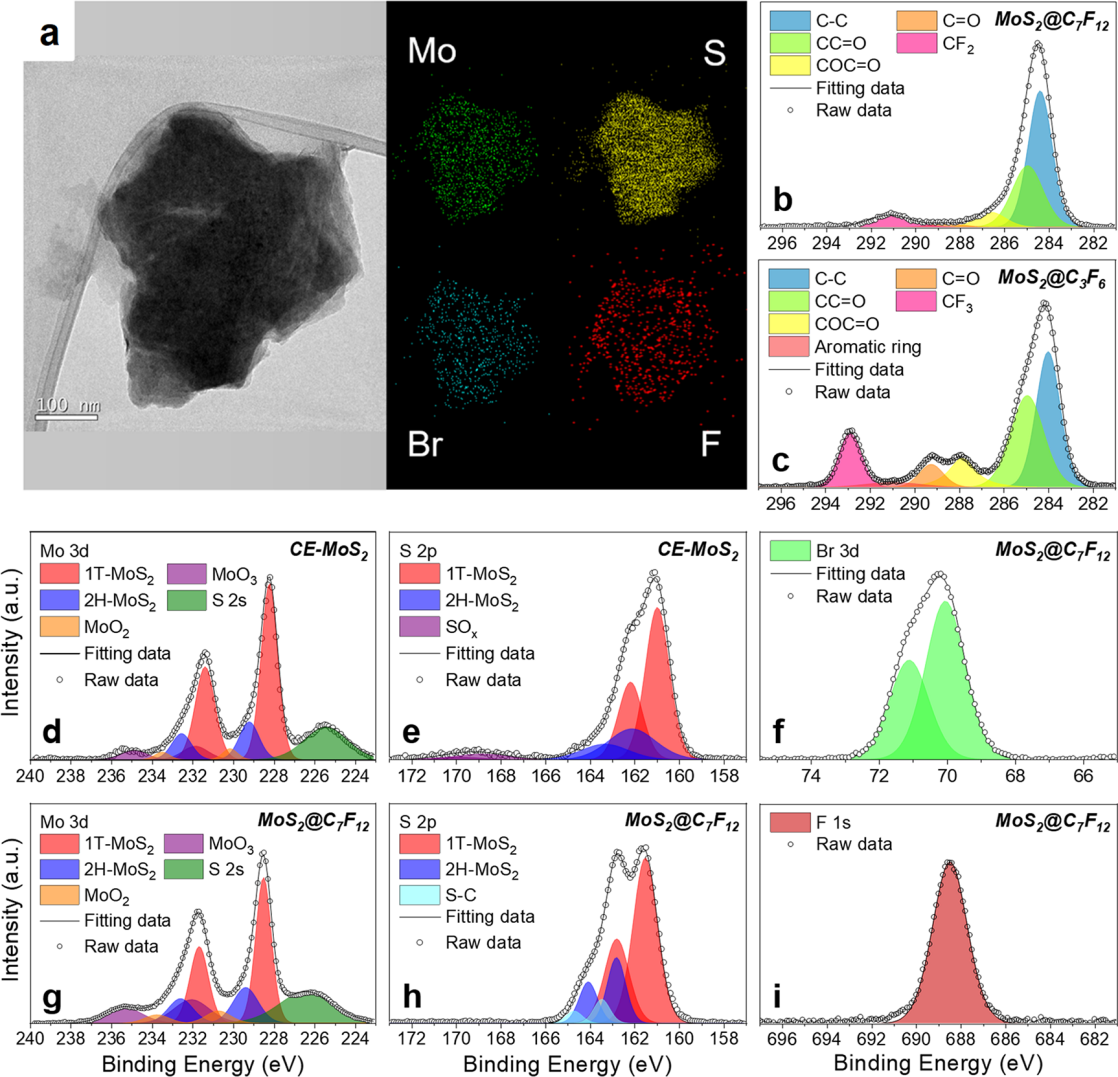
(a) HR-TEM images of a flake of functionalized MoS_2_@C_7_F_12_ complemented by EDX mapping of Mo, S, Br, and F (colored respectively in green, yellow, blue, and red). (b–i) XPS measurements of CE-MoS_2_ and functionalized MoS_2_@C_7_F_12_ and MoS_2_@C_7_F_12_.

The morphology of the functionalized MoS_2_@C_3_F_6_ and MoS_2_@C_7_F_12_ flakes was studied by means of HR-TEM as compared to the bare CE-MoS_2_ material (see Figure 3a and Figure S2). It is observed that MoS_2_ flakes preserve their characteristic 2D structure after functionalization; however, their tendency toward stacking/agreggating increases, hindering an efficient dispersion into single layers as deduced from AFM analysis (see Figure S11). This observation is likely due to the presence of new van der Waals interactions between the carbon chains. The thickness of the polymer shell was estimated to be 1–2.5 nm for the polymerization of the two different monomers, C_3_F_6_ and C_7_F_12_. These values were extracted by AFM analysis of the different flakes height profiles performed before and after surface functionalization (Figure S12). The chemical composition of the coated flakes was studied by energy-dispersive X-ray (EDX) analysis, revealing the presence of the main functional groups, represented by bromine (from the diazonium molecule) and fluorine (from the acryl molecules). The distribution of these elements along the flake is homogeneous, indicating the formation of a uniform coating.

The electronic signature and the composition of the MoS_2_@C_3_F_6_ and MoS_2_@C_7_F_12_ coated flakes were studied by X-ray photoelectron spectroscopy (XPS). Given the comparable results obtained for the two materials, only the composite MoS_2_@C_7_F_12_ is discussed below (Figure 3b–i), whereas details for MoS_2_@C_3_F_6_ XPS spectra are described in Figure S13. Essentially, clear differences were observed between the polymer-coated and the control CE-MoS_2_ materials. It is important to remark that MoS_2_ can present two main polytypes, i.e., a hexagonal 2H phase and the tetragonal 1T phase. The former phase is more abundant in MoS_2_ bulk crystals or mechanically exfoliated layers, whereas the latter is commonly obtained after chemical exfoliation processes, as in the present study. Focusing on the Mo 3d XPS spectra, the predominance of the 1T phase can be discerned in CE-MoS_2_, with peaks at ∼228.2 eV for Mo 3d_5/2_ and ∼231.4 eV for Mo 3d_3/2_ (Figure 3d). After functionalization with acryl-C_7_F_12_, the same 1T phase predominance is maintained with peaks moving to ∼228.5 and ∼231.7 eV, respectively, for Mo 3d_5/2_ and Mo 3d_3/2_ (Figure 3g). Such blue-shifting is likely related to the loss of electron density at the CE-MoS_2_ interphase, produced by the electron transfer between the electron-rich metallic 1T-MoS_2_ and the bromobenzene diazonium salt to form the aryl radicals. This phenomenon is also evidenced in the S 2p spectrum (Figure 3e, h), which is moreover accompanied by the appearance of a brand-new doublet with S 2p_3/2_ at ∼163.5 eV and S 2p_1/2_ at ∼164.8 eV. This doublet is typically assigned to the presence of S–C bonds,^18−20,40,41^ suggesting a covalent functionalization of MoS_2_, which corresponds to 7% based on sulfur in the case of MoS_2_@C_7_F_12_ and 5% for MoS_2_@C_3_F_6_, as expected for typical diazonium-based reactions.^19^ The presence of new bands in the carbon region is in good agreement with the anchored vinyl polymer, including the major groups CF_2_ and CF_3_ for MoS_2_@C_7_F_12_ and MoS_2_@C_3_F_6_, respectively (Figure 3b, c).^42^ In addition, the presence of the new F 1s band at 688.5 eV in the coated MoS_2_@C_7_F_12_ and MoS_2_@C_3_F_6_ endorses the existence of CF_2_ and CF_3_ groups from the fluorinated monomers (Figure 3i).^42^ Finally, in the Br 3d XPS region, the characteristic Br 3d doublet with the main peak at 70.0 eV (Br 3d_5/2_) accompanied by a 1.10 eV spin–orbit coupling can be seen, confirming the presence of bromobenzene in the coated materials (Figure 3f).

**Figure 4:**
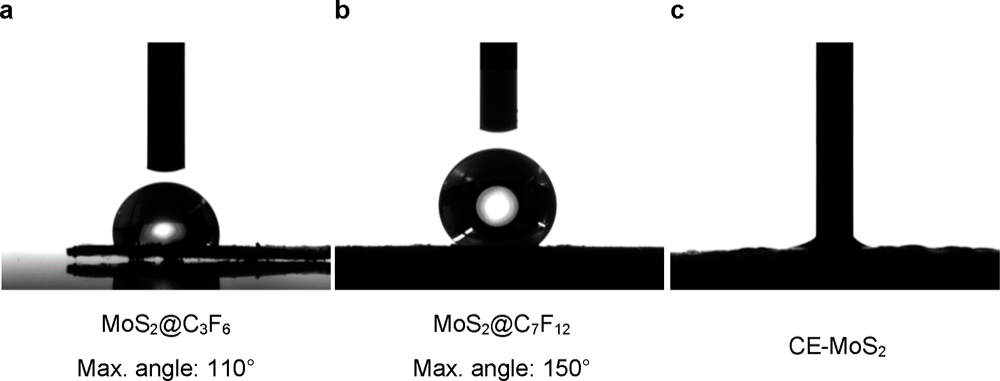
Images of water drops in contact with the surface of (a) MoS_2_@C_3_F_6_, (b) MoS_2_@C_7_F_12_, and (c) CE-MoS_2_ and the different contact angles that they exhibit.

Among the different functionalities that can be incorporated into the CE-MoS_2_ following this flexible vinylic polymerization reaction, hydrophobicity was first selected to clearly evidence the coating performance. Broadly, imparting surface hydrophobicity can be a key aspect of a 2D material system, which may prevent chemical instability, govern cell proliferation, improve antibacterial effects, or provide oil–water separation, among other characteristics.^43−47^ Coating performance was evaluated through contact angle measurements performed on pellets of the corresponding coated MoS_2_@C_3_F_6_ and MoS_2_@C_7_F_12_ materials and the unmodified CE-MoS_2_ (see Figure 4). In the case of the pristine CE-MoS_2_, the water drops completely spread over the surface because of the high hydrophilicity provoked by the exfoliation protocol (Figure 4c). In contrast, the contact angle value of the coated MoS_2_@C_3_F_6_ and MoS_2_@C_7_F_12_ was found of 110 and 150°, respectively (Figure 4a, b). These results evidence that MoS_2_ truly becomes highly hydrophobic when coated through in situ formation of fluorinated polymers. The larger angles obtained in the MoS_2_@C_7_F_12_ material suggest that hydrophobicity is drastically affected by the number of F atoms of the acryl molecules, resulting in more hydrophobicity when larger fluorinated acryl monomers are used. Deeper analysis of the polymerization will be required to evaluate this different hydrophobicity, as it is likely linked to the extent of dendritic structure formation. Finally, the advantage provided by the added surface hydrophobicity on MoS_2_ was evaluated by determining the chemical stability of the flakes in air. XPS measurements were performed on 7 month aged samples of CE-MoS_2_ and coated-MoS_2_ and compared to freshly prepared samples (Figure S14). A clear increase in the band corresponding to oxidized Mo(VI) species can be observed in CE-MoS_2_, whereas in the case of the functionalized MoS_2_@C_7_F_12_ and MoS_2_@C_3_F_6_ materials, this band remains practically constant. These results show the effective use of the developed hydrophobic functionalization as a protective coating for CE-MoS_2_ against oxidation, which could be extended to other more air-sensitive related 2D materials.

## Conclusions

We have successfully applied a diazonium anchoring reaction to provoke the covalent adhesion of functional polymeric coatings onto CE-MoS_2_ flakes. The reaction uses the mild diazonium chemical reduction upon electron transfer from the metallic 1T-MoS_2_ to form a first phenylene layer, which acts as the base for the radical growth of vinyl polymers formed in situ. In this work, functional acryl monomers comprising hydrophobic groups and varying chain lengths were selected. In both cases, the covalent coating occurred in a fast and simple reaction and was evidenced by multiple experimental techniques including TGA-MS and XPS. The coated materials exhibit large hydrophobic behavior arising from the anchored fluorinated molecules of the organic coating, as evidenced by contact angle measurements. Thanks to the coating, the stability of the 2D material has been improved, opening the door to its use in practical devices operating at ambient conditions. We anticipate that the reported chemical functionalization may be applied using practically any acrylate molecule to form functional polymeric coatings with strong interfacial bonding between the organic functional matrix and the MoS_2_, which significantly expands the possibilities of the 2D material for numerous applications.

## Experimental Section

### Materials

All chemicals involved in the exfoliation and functionalization of MoS_2_, including organic solvents, are commercially available and were used as received without additional purification. *n*-Butyllithium (1.6 M in hexane), 1,1,1,3,3,3-hexafluoroisopropyl acrylate (99%), 2,2,3,3,4,4,5,5,6,6,7,7-dodecafluoroheptyl acrylate (95%), and 4-bromobenzenediazonium tetrafluoroborate (96%) were purchased from Sigma-Aldrich. Molybdenum(IV) sulfide powder was obtained from Alfa Aesar. Acetonitrile was purchased from Honeywell and Milli-Q water was obtained from a Millipore Milli-Q system.

### Chemical Exfoliation of MoS_2_

Chemically exfoliated 1T-MoS_2_ was obtained following a previously reported method.^12^ A sealed Teflon autoclave reactor containing polycrystalline commercial MoS_2_ powder (320 mg, 2 mmol) and *n*-butyllithium (5 mL, 8 mmol) was heated inside an oven at 100 °C for 2 h. After that, the intercalation product was retrieved by filtration under nitrogen. After exposing the black solid (∼300 mg) to air and mixing it with degassed Milli-Q water (10 mL), it was dispersed in an ultrasonic bath sonicator for 1 h. The resultant dispersion was dialyzed for 16 h, transferred into a centrifuge tube, bath-sonicated for 30 min, and finally centrifuged at 750 rpm for 30 min. The obtained 1T-MoS_2_ suspension was used without further purification.

### Covalent Functionalization of CE-MoS_2_

All functionalizations were carried out in flasks, and occurred under mild conditions, in open air and at room temperature. In a general procedure, an aqueous suspension of CE-MoS_2_ (0.1 mmol, 25 mM) was reacted with a freshly prepared solution of 4-bromobenzenediazonium (0.3 mmol, 60 mM) in acetonitrile. Rapidly, 1 mmol of the corresponding acryl-C_3_F_6_ (168 μL) or acryl-C_7_F_12_ (245 μL) molecules were directly added and the suspensions were maintained under stirring for several minutes. Immediately after the addition of the acryl monomers, a black flocculate formed in a quantitative manner. The coated material was collected by centrifugation (5 min, 1500 rpm) and thoroughly washed with portions of 10 mL of acetonitrile (×3) to ensure the removal of nonreacting products. The coated MoS_2_ materials were then dried under vacuum overnight for structural and morphological characterizations.

### Characterization

The materials were characterized by FTIR, Raman, UV–vis, and X-ray photoelectron (XPS) spectroscopies, atomic force (AFM) and high-resolution transmission electron (HR-TEM) microscopies, thermogravimetric analysis (TGA), TGA coupled to mass spectroscopy (TGA-MS), and laser desorption/ionization-time of flight (LDI-TOF). ATR-FTIR spectra were obtained using an ALPHA II FTIR spectrometer (Bruker) in the 4000–400 cm^–1^ range with a resolution of 4 cm^–1^ in the absence of KBr pellet. Raman spectra were recorded using a Horiba-MTB Xplora. All samples were measured under continuous-wave operation (CW), exciting the sample at 532 nm wavelength with 0.8 mW excitation power. Light was focused on the sample using a regular microscope objective (100× magnification, Olympus brand) with a working distance of 0.21 mm. Laser power was measured by placing a laser power meter (Maxlab-TOP from Coherent Inc.) below the objective. The UV–vis spectrum was recorded on a Jasco V-670 spectrophotometer in baseline mode from 190 to 1200 nm range, using a 1 cm optical path quartz cuvettes. XPS measurements were performed in a K-ALPHA Thermo Scientific equipment with an X-ray source of Al Kα radiation (1486.6 eV), monochromatized by a twin crystal monochromator. The samples were drop-casted on gold-coated silicon substrates. The data were fitted with the *Avantage* software, using a smart background to approximate the experimental backgrounds, and spectra were referenced using the Au 4f main peak (84.0 eV). For AFM measurements, the substrates were imaged with a Digital Instruments Veeco Nanoscope IVa AFM microscope in tapping mode, using silicon tips with a resonance frequency of 300 kHz and with an equivalent constant force of 40 N m^–1^. AFM images were treated with Gwyddion. HR-TEM images were obtained using a TECNAI G2 F20 (FEI), which permitted energy-dispersive X-ray (EDX) mapping. TEM samples were prepared by drop-casting sample solutions on the ultrathin carbon mesh copper grids and dried under ambient conditions. TGA was performed using a TGA 550 (TA Instruments) at a heating rate of 5 °C min^–1^ from 25 to 600 °C under nitrogen. TGA-MS measurements were made in a NETZSCH STA 449F5 instrument under an argon atmosphere in the 40–800 °C range at a heating rate of 5 °C min^–1^. The composition of the samples was further analyzed using a 5800 MALDI TOF (ABSciex) instrument operating in a mass range of 300–2000 *m*/*z* in reflector positive mode, with a laser intensity of 4500.

#### Contact Angle Experimental Details

Dynamic water contact angle measurements of the samples were performed in air using a Ramé-hart 200 standard goniometer equipped with an automated dispensing system. Aliquots of deionized water were consecutively added to a pellet of the different coated MoS_2_@C_3_F_6_ and MoS_2_@C_7_F_12_ flakes as compared to the CE-MoS_2_ material, and the contact angle of the drop with the surface was measured. The obtained contact angle values resulted from a combination of three cycles, each cycle comprising 15 successive new additions of deionized water aliquots. For each cycle, the initial drop volume was 1 μL, followed by additions of 0.25 μL and waiting times of 1000 ms for each step.

## Supporting Information Available

General characterization of MoS_2_ (UV/vis, HRTEM, AFM, and TGA) and detailed characterization of the functionalized materials (IR, Raman, MALDI-TOF, and XPS) (PDF)
