# Supplementary material for: Fast Polymeric Functionalization Approach for the Covalent Coating of MoS2 Layers
Source: ACS Appl Mater Interfaces. 2021 Jul 23;13(30):36475–81. doi: 10.1021/acsami.1c08294 (PMC9127790; doi:10.1021/acsami.1c08294)
Supplement: Supplementary file 1 — am1c08294_si_001.pdf [file am1c08294_si_001.pdf]

Supporting Information

Fast polymeric functionalization approach for the covalent coating of MoS_2 layers

*Iván Gómez-Muñoz, Sofiane Laghouati, Ramón Torres-Cavanillas, Marc Morant-Giner, Natalia V. Vassilyeva, Alicia Forment-Aliaga, Mónica Giménez-Marqués**

Instituto de Ciencia Molecular (ICMol), Universidad de Valencia, c/Catedrático José Beltrán 2, Paterna, 46980, Spain.

KEYWORDS: 2D materials; transition metal dichalcogenides; covalent functionalization; diazonium chemistry; surface polymerization.

1. **Characterization of CE-MoS_2**

Figure S1. UV/Vis spectroscopy

Figure S2. HRTEM image and AFM topographic image

Figure S3. Thermogravimetric analysis of CE-MoS_2

2. **Characterization of MoS_2@C_3F_6 and MoS_2@C_7F_{12}**

Figure S4. Picture of CE-MoS_2 and coated MoS_2@C_3F_6 aqueous suspensions

Figure S5-S6. Infrared spectroscopy

Figure S7. Raman spectroscopy

Figure S8. Thermogravimetric analysis of C_3F_6 and C_7F_{12}

Figure S9-S10. Mass spectrometry (MS) characterization

Figure S11-S12. AFM images

Figure S13-S14. X-ray photoelectron spectroscopy

1. Characterization of CE-MoS_2

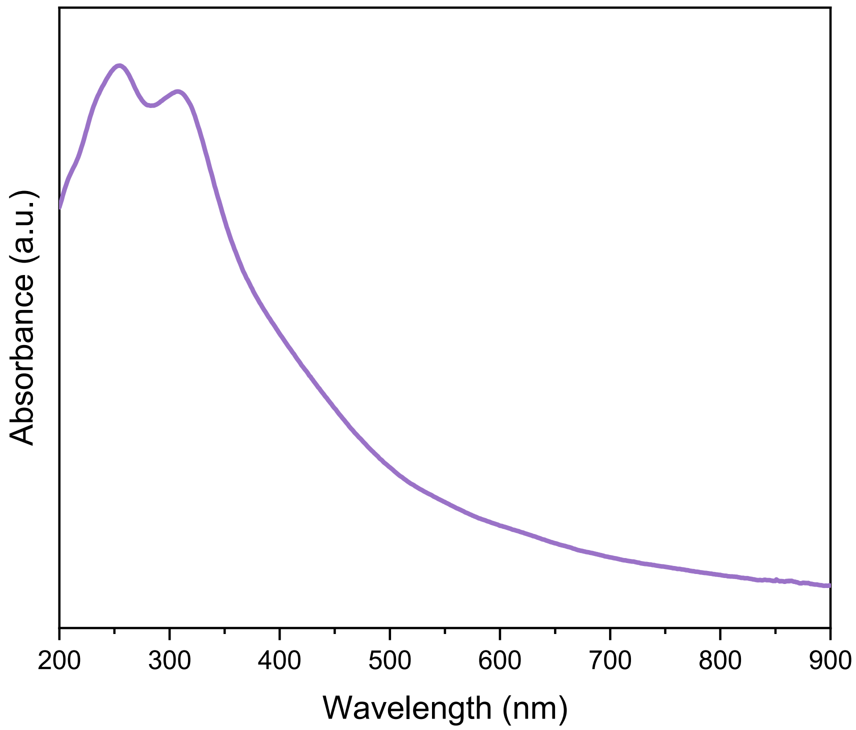

Figure S1. UV/Vis spectrum of a ≈0.01 mg/mL suspension of CE-MoS_2 flakes in water, exhibiting the two characteristic bands of 1T-MoS_2 at 255 and 307 nm.[2]

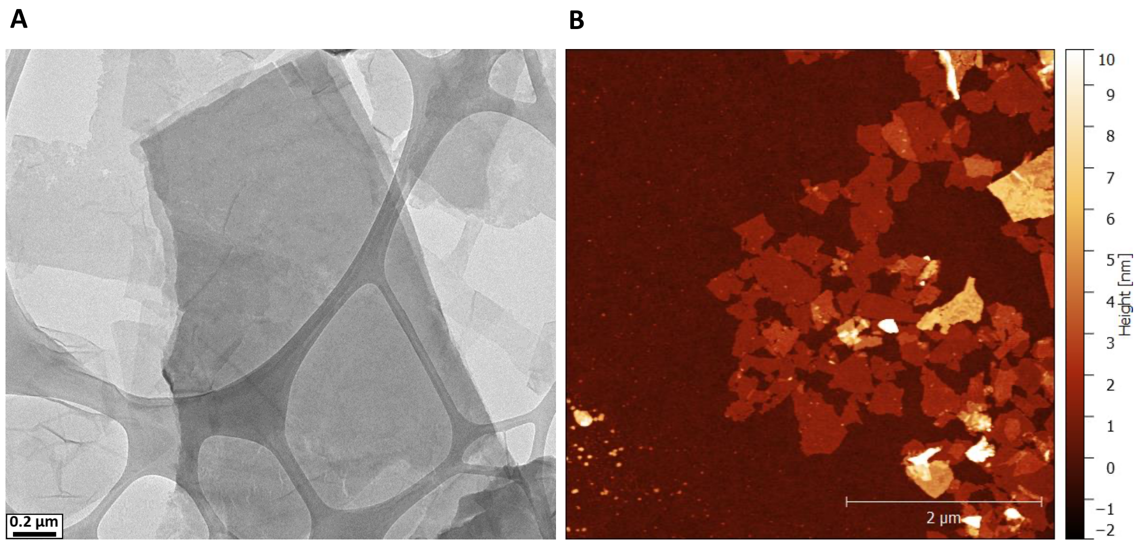

Figure S2. A) HRTEM images and B) AFM topographic image of CE-MoS_2, where thin flakes can be observed

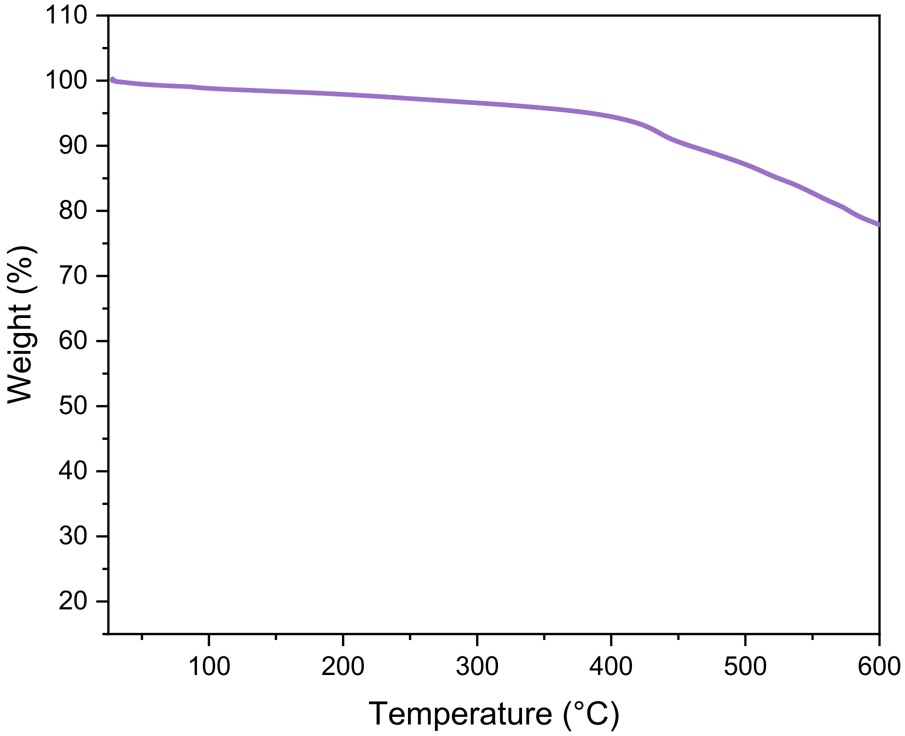

Figure S3. Thermogravimetric analysis of CE-MoS_2 showing that the material is thermally stable until 400 °C. The analysis was performed at a heating rate of 5 °C/min from 25-600 °C under nitrogen atmosphere.

2. Characterization of $MoS_2@C_3F_6$ and $MoS_2@C_7F_{12}$

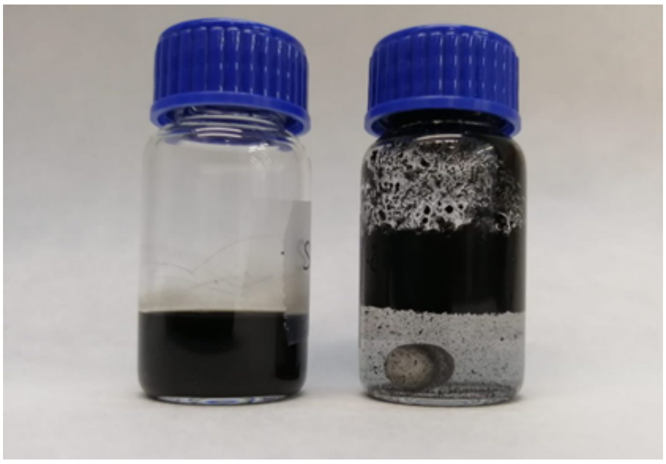

Figure S4. Picture of CE-MoS_2 aqueous suspensions before (left) and after (right) functionalization. The hydrophobic character endowed upon functionalization in $MoS_2@C_3F_6$ is clearly identified by the formation of a flocculate in water.

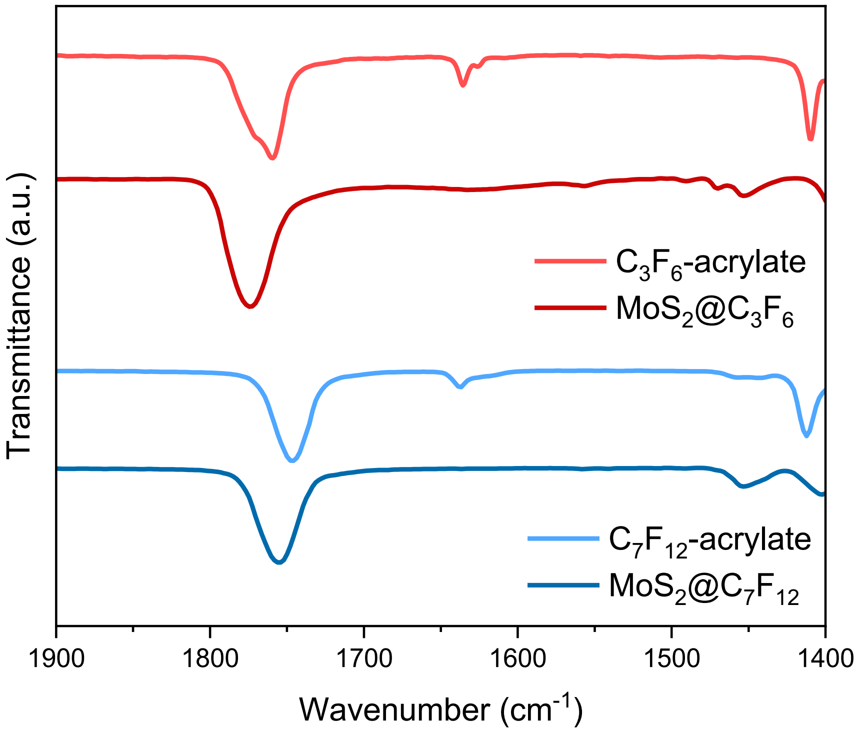

Figure S5. Infrared spectra of functionalized $MoS_2@C_3F_6$ (dark red) and $MoS_2@C_7F_{12}$ (dark blue) materials as compared with the corresponding commercial acrylate molecules (light colors). It is observed that the characteristic C=C stretching band of the acrylate molecules located at 1635 cm^{-1} disappears upon functionalization, which is accompanied by the corresponding blue-shift of the C=O stretching band, in agreement with a grafting reaction.

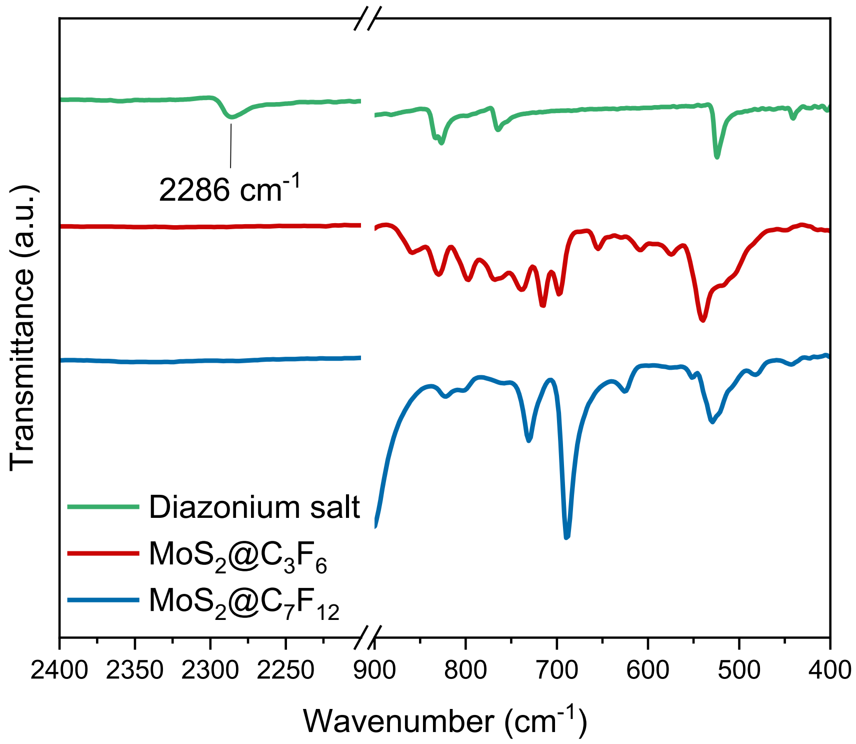

Figure S6. Infrared spectra of functionalized $MoS_2@C_3F_6$ (red) and $MoS_2@C_7F_{12}$ (blue) materials as compared with the 4-bromobenzene diazonium salt (green). The absence of the characteristic N-N stretching at 2286 cm^{-1} in the functionalized materials, which is attributed to the N_2^+ group of the diazonium salt, confirms the anchoring of the phenylene to the surface.[3]

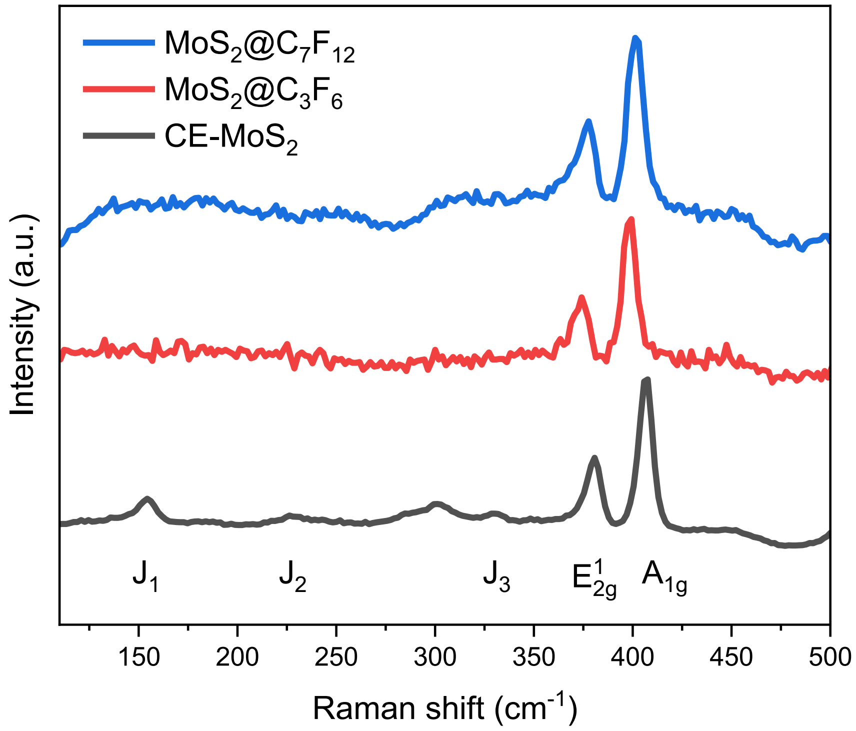

Figure S7. Raman spectra of CE-MoS_2 (gray), and different coated materials MoS_2@C_3F_6 (red), and MoS_2@C_7F_{12} (blue). The characteristic J_1 (154.4 cm^{-1}), J_2 (226.3 cm^{-1}), and J_3 (330.3 cm^{-1}) peaks disappear after the functionalization.

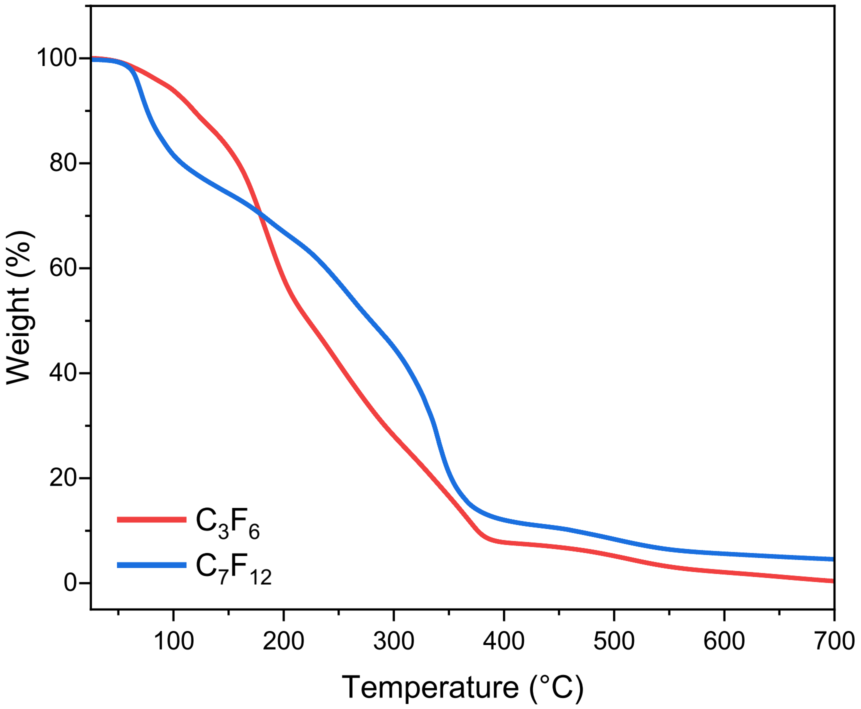

Figure S8. Thermogravimetric profiles of the different polymers synthesized from polymeric reaction of 4-bromobenzene diazonium salt with C_7F_{12} (blue line) and C_3F_6 (red line) in solution and in the absence of a 2D support.

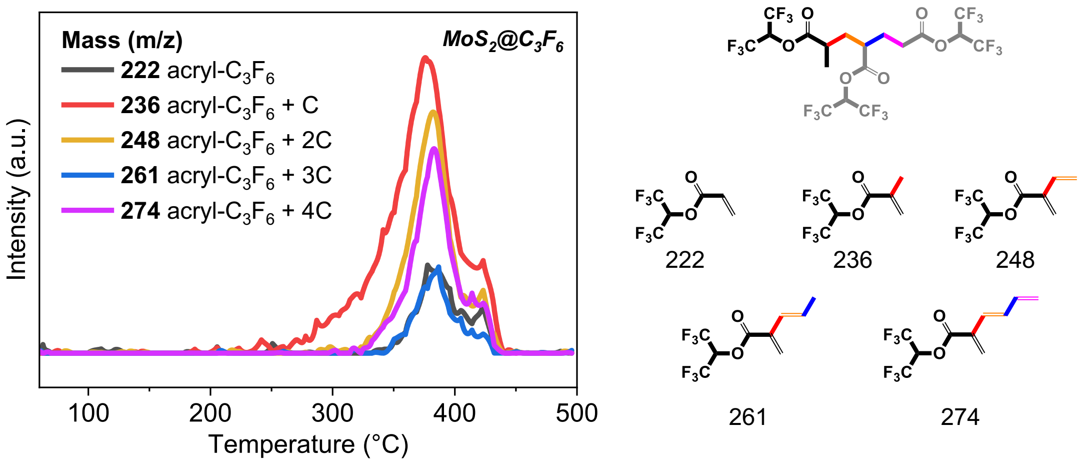

Figure S9. Proposed fragmentation of a vinyl-C_3F_6 trimer based on the mass losses of $MoS_2@C_3F_6$. Mass m/z = 222, which corresponds to the vinyl monomer (black), is followed by successive additions of one carbon-chain fragment depicting the chain structure of the vinyl-C_3F_6 trimer, as it is represented with the corresponding colours.

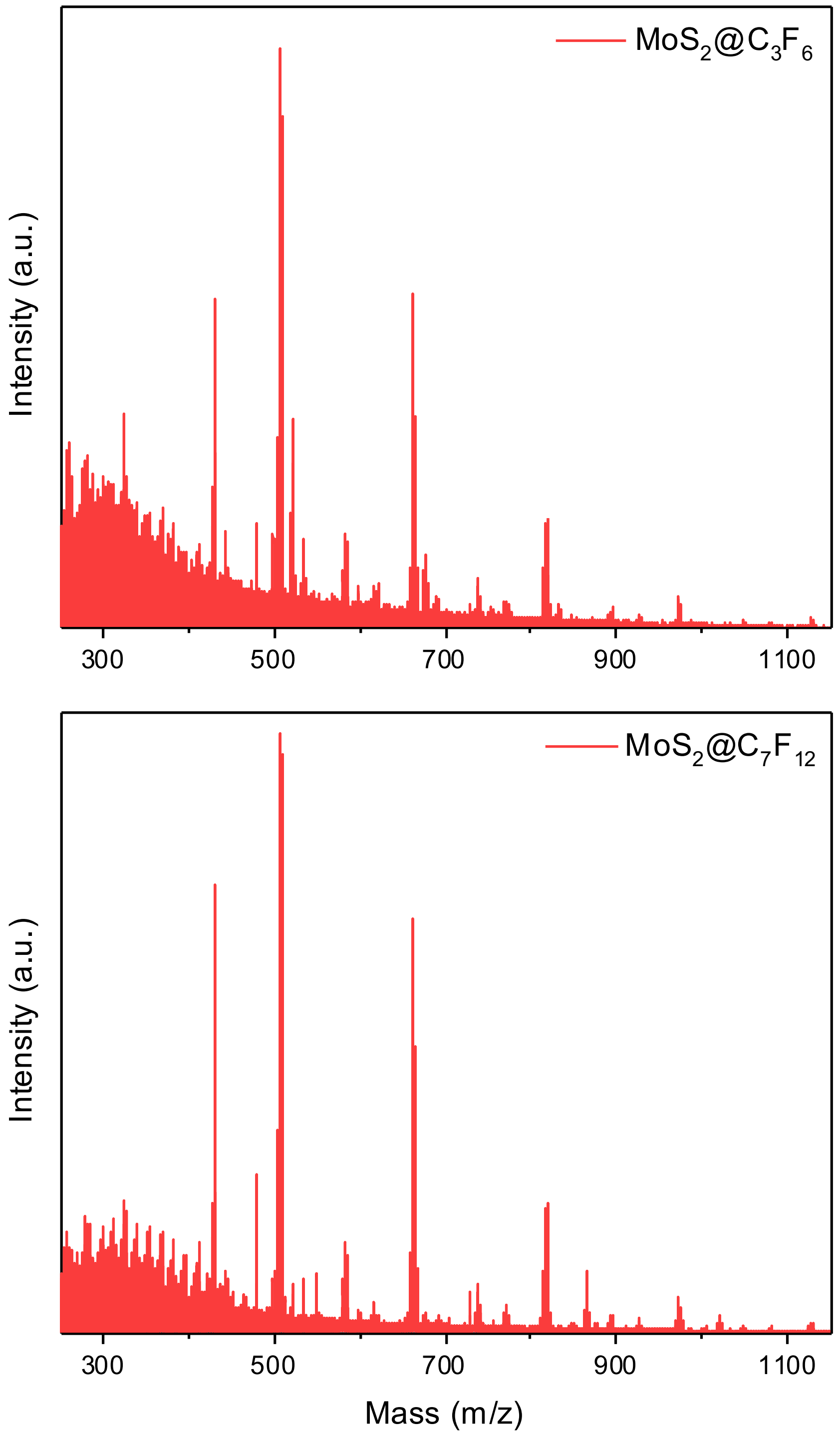

Figure S10. MALDI-TOF spectra of $MoS_2@C_3F_6$ (top), and $MoS_2@C_7F_{12}$ (bottom). In both samples, the base peak (BP) corresponds to 506.8 m/z, assigned to three covalently bonded bromoaryl molecules.

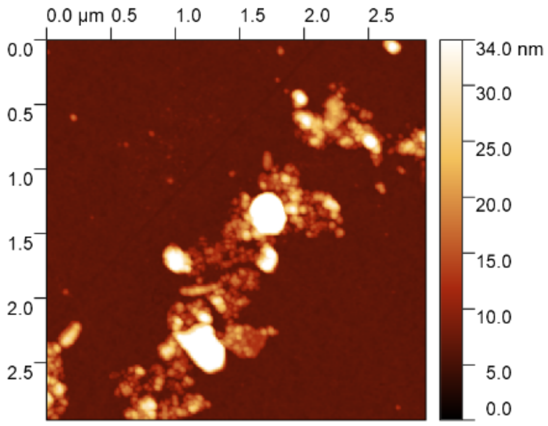

Figure S11. AFM image of $MoS_2@C_7F_{12}$ spin coated on a Si/SiO_2 (285 nm) substrate. The presence of large aggregates can be observed, hindering the possibility of further analysis.

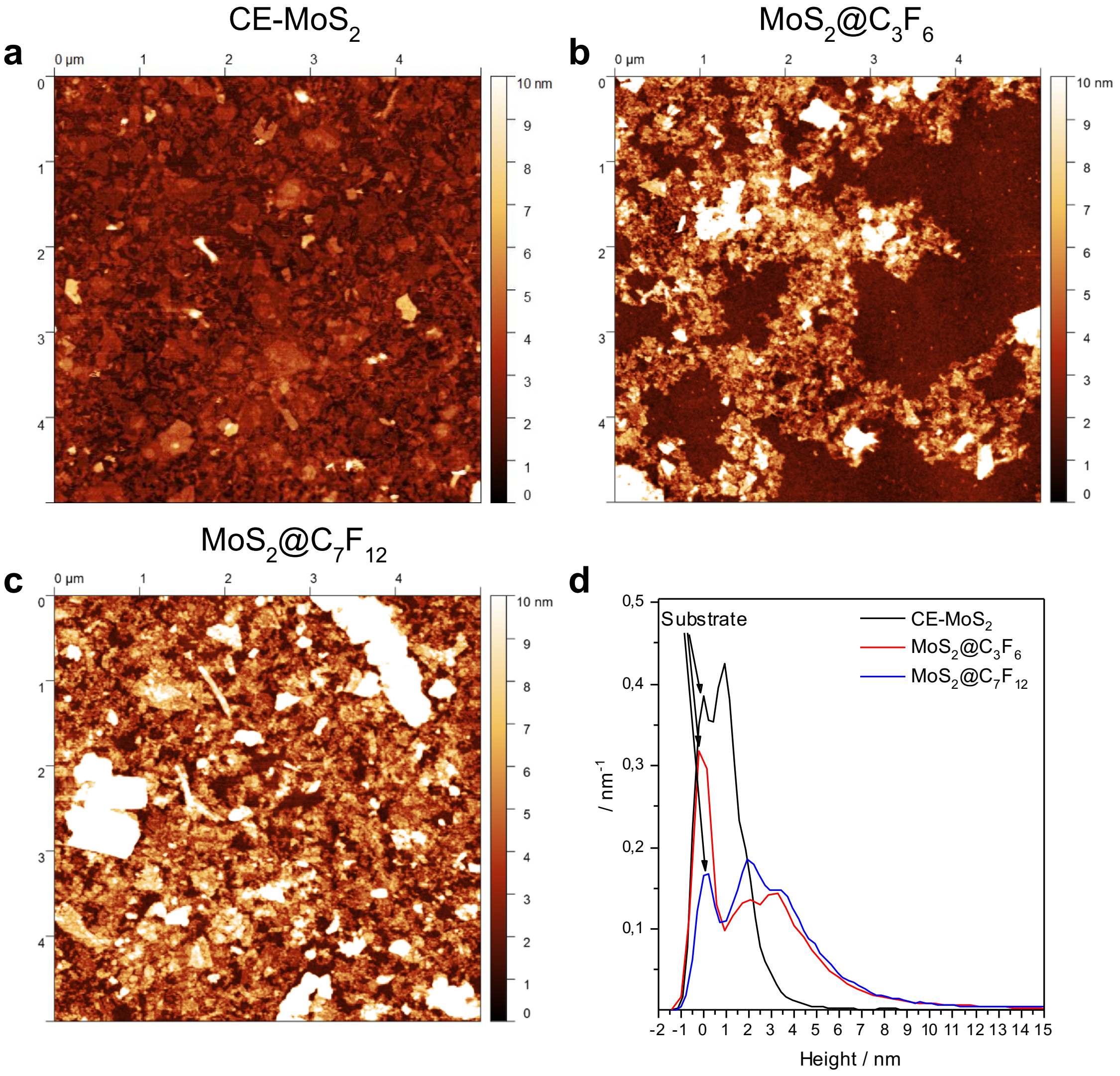

Figure S12. a) AFM images of bare CE-MoS_2 and functionalized b) MoS_2@C_3F_6 and c) MoS_2@C_7F_{12}. d) Height profile extracted from AFM images of CE-MoS_2 (black profile) spin-coated on a Si/SiO_2 (285 nm) substrate and analogous substrates immersed in aryl diazonium salt and acrylate C_3F_6 (red profile) or C_7F_{12} (blue profile) solutions to functionalize the flakes.

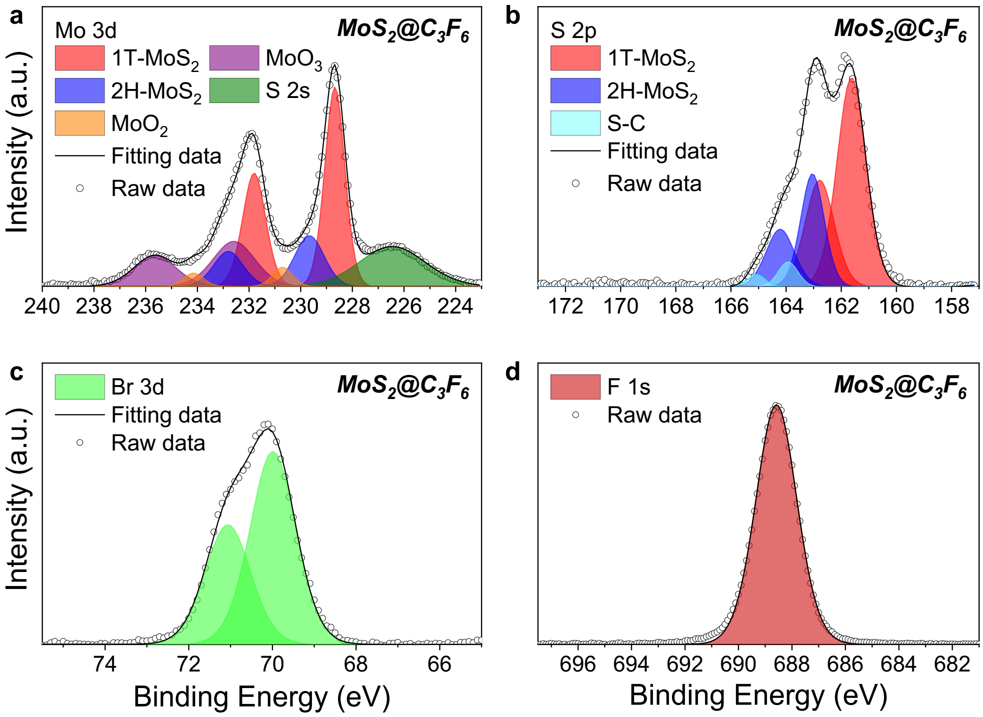

Figure S13. a) Mo 3d, b) S 2p, c) Br 3d, and d) F 1s X-ray photoelectron spectra of $MoS_2@C_3F_6$. The predominance of the 1T phase is maintained after the functionalization with a blue-shitfing to Mo $3d_{5/2}$ = 228.7 eV and $3d_{3/2}$ = 231.8 eV, possibly related to the electron transfer between the electron-rich 1T phase to the bromobenzene diazonium salt. 5% of grafting per sulfur atom is suggested with the peaks at S $2p_{3/2}$ at 163.9 eV and $2p_{1/2}$ at 165 eV, attributed to S-C. The spectra of Br 3d (c) and F 1s (d) confirm the presence of bromobenzene and the fluorinated polymer in the coated materials.

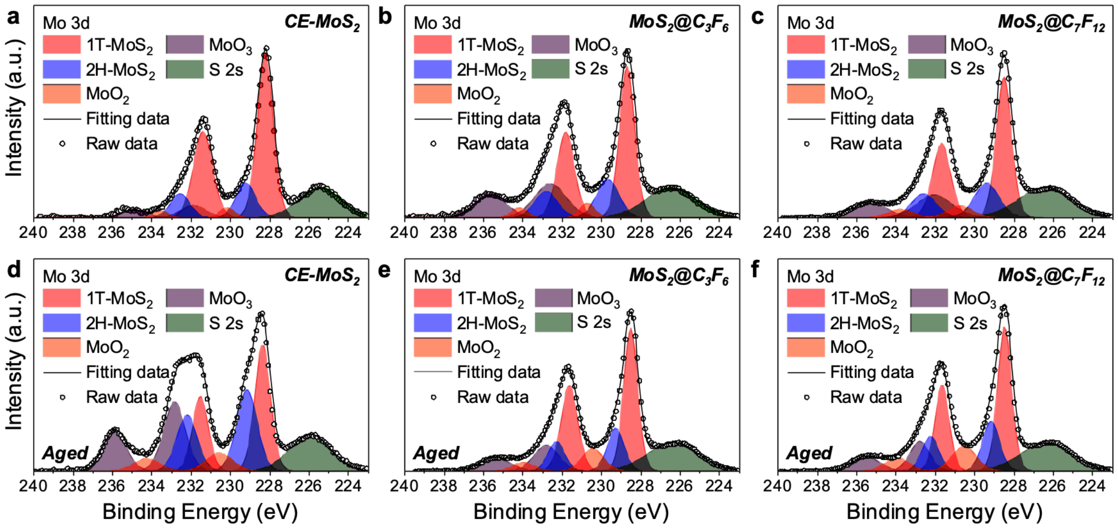

Figure S14. Mo 3d X-ray photoelectron spectra of freshly prepared (a,b,c) and 7 months-aged samples of bare CE-MoS_2 (a, d), and coated $MoS_2@C_3F_6$ (b, f) and $MoS_2@C_7F_{12}$ (c, g) materials.

References:

(1) Morant-Giner, M.; Sanchis-Gual, R.; Romero, J.; Alberola, A.; García-Cruz, L.; Agouram, S.; Galbiati, M.; Padial, N. M.; Waerenborgh, J. C.; Martí-Gastaldo, C.; Tatay, S.; Forment-Aliaga, A.; Coronado, E. Prussian Blue@MoS_2 Layer Composites as Highly Efficient Cathodes for Sodium- and Potassium-Ion Batteries. *Adv. Funct. Mater.* **2018**, *28* (27), No. 1706125.

(2) Voiry, D.; Goswami, A.; Kappera, R.; Silva, C. D. C. C. E.; Kaplan, D.; Fujita, T.; Chen, M.; Asefa, T.; Chhowalla, M. Covalent Functionalization of Monolayered Transition Metal Dichalcogenides by Phase Engineering. *Nat. Chem.* **2015**, *7* (1), 45-49.

(3) Chu, X. S.; Yousaf, A.; Li, D. O,; Tang, A. A.; Debnath, A.; Ma, D.; Green, A. A.; Santos, E. J. G.; Wang, Q. H. Direct Covalent Chemical Functionalization of Unmodified Two-Dimensional Molybdenum Disulfide. *Chem. Mater.* **2018**, *30*, 2112-2128.
